# Hospital Benefit and the Insurance Act

**Published:** 1912-01-13

**Authors:** 


### HOSPITAL BENEFIT AND THE INSURANCE ACT.

By Thirty Years a Hospital Superintendent.

Sir,—Having seen a copy of the optimistic views expressed by Mr. Michelli, who has probably the most popular object to appeal for of any secretary, I desire to put in a few words of warning. Mr. Michelli has evidently no knowledge of the circumstances which affect great provincial, Scottish, and other hospitals. He assumes that each Voluntary Hospital can permit greater abuses to be introduced into its in-patient department through the Insurance Act than any which have ever prevailed among the out-patients. I know from the views expressed to me by some of the most generous contributors to the Voluntary Hospitals that any hospital which permits such in-patient abuse will lose largely and properly in income. Mr. Michelli ignores the evils threatened by the Insurance Act, which are that the breadwinner—that is, the man who is now paying for adequate medical service for himself, which he is promised under the Bill—may or will not be able to obtain hospital care. This is so because:—

### Why Hospital Care may not be Obtainable.

(1) The withdrawal of subscriptions means the diminution of beds, and there are not enough hospital beds at present for the requirements of our breadwinners. Several large hospitals have long lists of patients waiting to be admitted.

(2) The workman will be ineligible for free, that is charitable relief at a Voluntary Hospital as an insured person, for Voluntary Hospitals under their constitution provide for those breadwinners who, when well, are able to maintain themselves and their dependents, but who, however, cannot defray the cost of medical and hospital treatment when ill.

(3) The working classes are to enjoy rare and refreshing fruit in the shape of adequate medical treatment under the National Insurance Act free from the taint of charity. A workman may have voluntarily contributed a penny or twopence a week to the Voluntary Hospitals, and has always had adequate hospital benefit when he required it. Now, under the Act, he is compulsorily made to pay 4d. a week, and may never be able to obtain, as the Act stands, the hospital benefit he may require when seriously ill.

(4) The Insurance Act is stated by its author to be based upon the German Acts. Adequate medical relief under the German Acts includes hospital treatment. Unless hospital treatment is included under the National Insurance Act in this country it does not fulfil the claims of its author, and must place breadwinners all over the country in a most cruel position in the hour when they are most grievously ill. If a voluntary hospital were to admit and treat thousands of insured persons as in-patients, and to give them free medical relief, that would cause the in-patient department to be subjected to graver abuses than any which have yet occurred in connection with our hospitals. Directly a hospital was known by the benevolent to be sanctioning these abuses it is fair to assume that the contributions of a large number of its most liberal supporters would be withdrawn.

(5) British workmen are certainly not going to agree quietly to receive less than the German workmen when they pay their fourpences. The German workman gets hospital benefit for his compulsory payments, and I do not believe my fellow countrymen who are working men and breadwinners will rest content until justice is done them, by the passing of an amending Act, before the present National Insurance Act comes into force, which will secure to them hospital benefit as and when they may require it.

### In a Nutshell.

(6) The position in a nutshell is this. The National Insurance Act cuts right across the sources of supply of money to the Voluntary Hospitals, which in the past have provided all the hospital care required by our breadwinners. The danger of the absence of sufficient hospital beds for the care and cure of our breadwinners should be remedied by the passing of an amending Act, which, on a *pro rata* basis, will enable the Insurance Commissioners to make contracts with those Voluntary Hospital managers who are prepared to admit State cases. The basis is that the Commissioners will pay to the hospitals the actual cost of each patient resident, plus 10 per cent. for the remuneration of the doctors engaged. In this way justice will be done all round and the voluntary principle of our hospitals will be safeguarded. N.

